# In vivo vesicular acetylcholine transporter density in human peripheral organs: an [^18^F]FEOBV PET/CT study

**DOI:** 10.1186/s13550-022-00889-9

**Published:** 2022-04-01

**Authors:** Jacob Horsager, Niels Okkels, Nathalie Van Den Berge, Jan Jacobsen, Anna Schact, Ole Lajord Munk, Kim Vang, Dirk Bender, David J. Brooks, Per Borghammer

**Affiliations:** 1 Department of Nuclear Medicine and PET, Aarhus University Hospital, Palle Juul-Jensens Boulevard 165, J220, 8200 Aarhus N, Denmark; 2 Department of Clinical Medicine, Aarhus University, Aarhus, Denmark; 3 Department of Neurology, Aarhus University Hospital, Aarhus, Denmark; 4 Institute of Translational and Clinical Research, University of Newcastle Upon Tyne, Newcastle upon Tyne, UK

**Keywords:** VAChT, Vesicular acetylcholine transporter, Cholinergic neurons, Parasympathetic nervous system, PET imaging, [18F]FEOBV

## Abstract

**Background:**

The autonomic nervous system is frequently affected in some neurodegenerative diseases, including Parkinson’s disease and Dementia with Lewy bodies. In vivo imaging methods to visualize and quantify the peripheral cholinergic nervous system are lacking. By using [^18^F]FEOBV PET, we here describe the peripheral distribution of the specific cholinergic marker, vesicular acetylcholine transporters (VAChT), in human subjects. We included 15 healthy subjects aged 53–86 years for 70 min dynamic PET protocol of peripheral organs. We performed kinetic modelling of the adrenal gland, pancreas, myocardium, renal cortex, spleen, colon, and muscle using an image-derived input function from the aorta. A metabolite correction model was generated from venous blood samples. Three non-linear compartment models were tested. Additional time-activity curves from 6 to 70 min post injection were generated for prostate, thyroid, submandibular-, parotid-, and lacrimal glands.

**Results:**

A one-tissue compartment model generated the most robust fits to the data. Total volume-of-distribution rank order was: adrenal gland > pancreas > myocardium > spleen > renal cortex > muscle > colon. We found significant linear correlations between total volumes-of-distribution and standard uptake values in most organs.

**Conclusion:**

High [^18^F]FEOBV PET signal was found in structures with known cholinergic activity. We conclude that [^18^F]FEOBV PET is a valid tool for estimating VAChT density in human peripheral organs. Simple static images may replace kinetic modeling in some organs and significantly shorten scan duration.

*Clinical Trial Registration* Trial registration: NCT, NCT03554551. Registered 31 May 2018. https://clinicaltrials.gov/ct2/show/NCT03554551?term=NCT03554551&draw=2&rank=1.

**Supplementary Information:**

The online version contains supplementary material available at 10.1186/s13550-022-00889-9.

## Introduction

In vivo imaging of the noradrenergic sympathetic nervous system is widely used in research, and constitutes a supportive diagnostic criterion in some neurodegenerative diseases, e.g., Parkinson’s disease and dementia with Lewy bodies (DLB) [1, 2]. Less attention has been paid to the peripheral cholinergic nervous system which is also affected in these diseases. For instance, up to 50% of preganglionic parasympathetic neurons in the dorsal motor nucleus of the vagus (DMV) are lost in deceased PD patients [3–5]. DLB patients show a similar degree of Lewy pathology in the DMV upon autopsy [6]. It has recently been hypothesized that the peripheral autonomic nervous system is affected at different time points in patients with PD and DLB [7]. Therefore, specific in vivo methods are needed to investigate the degree and timing of cholinergic degeneration in peripheral organs.

Previous studies have utilized the positron emission tomography (PET) tracer, 5-[^11^C]-methoxy-donepezil ([^11^C]donepezil), to measure acetylcholinesterase (AChE) density in visceral organs [8]. [^11^C]donepezil signal correlates well with known cholinergic innervation, but is not optimal for quantification of cholinergic neurons. First, [^11^C]donepezil binds to sigma-1 receptors with high affinity [9]. Second, AChE is also produced by non-cholinergic neurons, and is expressed in high concentrations in regions not suspected to have (intense) cholinergic innervation in the brain [10]. Finally, AChE activity may be influenced by changes in synaptic activity, which potentially confounds an estimate of cholinergic synaptic density [11].

The vesicular acetylcholine transporter (VAChT) is a glycoprotein closely associated with other cholinergic markers, i.e., choline acetyltransferase (ChAT), acetylcholine, and AChE [12]. It is located almost exclusively in cholinergic neurons with the highest concentration in terminals [12–14]. Immunohistochemistry studies have visualized VAChT in nerve terminals of many peripheral organs including the heart, gastrointestinal canal, adrenal medulla, prostate, pancreas, skeletal muscle motor endplate, salivary-, and lacrimal glands [15].

Vesamicol inhibits VAChT and is not displaced by acetylcholine suggesting that the vesamicol binding site is detached from the acetylcholine recognition site on VAChT [16, 17]. These conditions make the vesamicol binding site an ideal target for VAChT visualization. The vesamicol derivative, [^18^F]fluoroethoxybenzovesamicol ([^18^F]FEOBV) has been thoroughly validated for PET studies of the brain in rodents, monkeys, and humans [18–22]. [^18^F]FEOBV is a specific marker for VAChT, reflects the known cholinergic innervation in the brain, displays low interference with sigma- and dopamine receptors (unlike other vesamicol derivatives), can be displaced by “cold” FEOBV, and shows only modest defluorination [18, 19, 23]. To date, only one PET study has utilized [^18^F]FEOBV to visualize the peripheral cholinergic system in humans [24]. That study showed favorable tracer kinetics for estimating cholinergic activity of the myocardium. Other peripheral organs have not been investigated.

In the present study we evaluated human in vivo VAChT distribution in 13 peripheral organs using a 70 min dynamic [^18^F]FEOBV PET/CT protocol.

## Methods

### Study group

We included 15 healthy controls (7 females), mean age 72.4 years (range 53–86 years). Exclusion criteria were: diabetes, neurological, psychiatric, gastrointestinal, or other serious diseases, previous cancer, major surgery or radiation therapy to the head, thorax, or abdomen. All participants provided written informed consent. The study was approved by the Science Ethical Committees of the Central Denmark Region (project number 1-10-72-201-18).

### [^18^F]FEOBV PET/CT protocol

[^18^F]FEOBV tracer preparation is described in Additional file 1: Material. All scans were performed on the same Siemens Biograph Vision 600 PET/CT scanner (*Siemens Healthcare, Erlangen, Germany*). Participants were placed in supine position with hands above their head. After a topogram and a CT scan for attenuation correction, approximately 200 MBq [^18^F]FEOBV was administered into a cubital vein. Simultaneously, the PET acquisition was initiated. First, 6 min dynamic PET was acquired with the 26 cm field-of-view covering the top of the heart and the upper part of the abdomen. Then, from 6 to 70 min, dynamic whole-body PET data were acquired using continuous bed motion divided into 7 × 2 min passes followed by 9 × 5 min passes. The total scan time adds up to 70 min due to inter-pass bed motion. Each frame length was adjusted to each individual based on height, i.e., tall subjects required faster bed motion and vice versa (range of total scan time: 68.1–73.4 min). After PET acquisition, an ultra-low-dose whole-body CT scan was performed, and subsequently an additional low-dose CT scan of the thorax and abdomen after intravenously administered CT-contrast enhancement. All PET images were reconstructed using TrueX + TOF, 4 iterations, 5 subsets, 440 matrix, 2-mm Gaussian filtering, relative scatter correction, attenuation correction and were decay-corrected back to injection time. The PET image voxel size was 1.65 × 1.65 × 3.0 mm^3^.

### Volume-of-interest definition

Time-activity curves were extracted from the dynamic PET images using different volume-of-interest (VOI) approaches. The whole-blood time-activity curve was acquired with a circular region-of-interest (ROI) with a diameter of 10 mm, placed in the center of the descending aorta on fused PET/CT images on 20 adjacent axial slices above the diaphragm. The colonic signal was assessed through three steps: (1) a section of the transverse/descending colon was outlined on the CT image; (2) this VOI was used as template for outlining the colonic PET-signal (great care was taken not to include spill-in from surrounding organs); (3) the PET VOI was normalized to the CT-derived anatomical colonic volume. This volume was corrected for air content by subtracting an isocontour VOI of -300 Hounsfield Units. The left adrenal, the submandibular and parotid glands were acquired by outlining the PET signal with subsequent normalization to the anatomical volume measured on the CT image. The liver and spleen VOIs were 8 mm wide and paralleled the organ border. Renal cortex, left ventricular myocardium, and pancreas were also sampled in this way with the VOIs placed on six adjacent slices. The pancreas VOI was placed in the hottest area in the center of the body and tail. Due to bile secretion, meaningful data could not be obtained from the head of pancreas. Circular muscle ROIs of 10 mm were placed bilaterally in the back muscles on 20 adjacent slices, approximately 30 mm lateral to the spinous process at the level of the diaphragm. Small circular ROIs were placed on six adjacent slices in prostate (diameter 15 mm) and in each thyroid lobule (diameter 6 mm). We abstained from uterus analysis because the signal was highly influenced by spillover of bladder and small intestinal signal. Finally, a circular ROI (diameter 6 mm) was centered in the hottest area of the lacrimal gland on two adjacent slices.

VOIs sampling aortic lumen, myocardium, skeletal muscle, pancreas, colon, adrenal gland, spleen, liver, renal cortex, and prostate were defined on the PET image fused to the low-dose contrast-enhanced CT scan of the abdomen and thorax, with a voxel size of 1 × 1 × 2 mm^3^. VOIs sampling submandibular, parotid, and lacrimal glands and the thyroid were placed on the PET fused to the ultra-low-dose whole-body CT scan with a subsequent voxel size of 1.5 × 1.5 × 5mm^3^. To reduce the influence of movement artefacts, all VOIs were adjusted to the PET signal separately on each time frame. All analyses were performed using PMOD 4.0 (*Zürich, Switzerland*). Examples of ROI-definitions for each organ are shown in Additional file 2: Fig. S1.

### Kinetic modelling

We did not measure arterial blood activity as an input function for the present study. Several steps were performed to yield the optimal image-derived input function. First, an image-derived arterial time-activity curve (TAC) was extracted from the PET/CT image using the aorta lumen VOI (see above). A similar approach has been validated for another [^18^F]tracer ([^18^F]FDG) with a close correlation between image-derived and arterial blood sample activity (Additional file 3: Fig. S2). Next, the fraction of plasma in whole blood (calculated from individual hematocrit measurements in venous blood), the fraction of [^18^F]FEOBV in plasma (i.e., not bound to red blood cells, see Additional file 1: Material), and metabolism of parent [^18^F]FEOBV, were measured. The fraction of [^18^F]FEOBV binding to plasma proteins could not be assessed because the tracer was almost entirely retained in the centrifugal filter. This occurred with [^18^F]FEOBV in blood, but also in a buffer solution. Thus, this fraction was conservatively set to 1 (no binding).

**Fig. 1:**
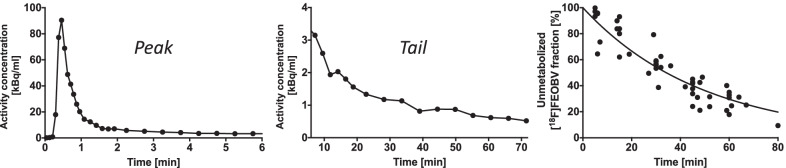
Example of an image-derived arterial plasma input function from aorta showing the initial peak (left), tail (middle), and the common metabolite correction curve (*Y* = 100% * e^(−0.2026*X)^) (right). The arterial plasma input function shown is corrected for hematocrit, red blood cell binding, and metabolites (see text for details)

Venous blood samples used for tracer metabolite correction were drawn during PET acquisition at approximately 5, 15, 30, 45, and 60 min post injection. In two subjects, it was not possible to draw cubital vein blood, when the arm was placed above the head inside the camera. This position, however, promotes higher quality images of the abdominal organs. 15 data points were excluded as they yielded parent fractions > 100% or increasing parent fraction over time—both situations are implausible. Also, very low counting numbers were observed during metabolite analysis procedure, which generated high statistical variation in the data. As a result, we chose to generate a population metabolite-correction curve derived for all 15 subjects (Fig. 1). Metabolites in venous blood samples exhibit a slight lag from those in arterial blood that was not corrected in the present study. This could have right-shifted the metabolite correction curve slightly, but the final curve was in close agreement with previously published data [20].

Kinetic analyses of the dynamic PET data could only be performed for organs with full time-activity curves (0–70 min), i.e., myocardium, pancreas, renal cortex, spleen, adrenal gland, muscle, and colon. We used three different non-linear approaches with the image-derived metabolite-corrected plasma TAC as input function: a 1-tissue compartment model (1TCM) with K_1_ and k_2_ as derived parameters; a reversible 2-tissue compartment model (2TCM) with K_1_, k_2_, k_3_, and k_4_ as derived parameters; and an irreversible 2TCM with K_1_, k_2_, and k_3_ as derived parameters (k_4_ constrained to 0). The blood volume fraction (V_0_) and time delay were derived in all fits. Vascular parameters were derived using uncorrected whole-blood TACs. The 1TCM performed best (see below), and the derived total volume-of-distribution (V_t_) was defined as K_1_/k_2_ [ml/ccm]. We also estimated V_t_ using Logan plots with estimated equilibrium times, a maximal error set to 10%, and with inclusion of a minimum of 6 data points (performed with the PKIN module in PMOD 4.0 *Zürich, Switzerland*). Here, the image-derived metabolite-corrected plasma TAC was used as the input function.

### Statistics

Normality was assessed with QQ-plots and histograms. The strength of the linear relationship between kinetic modeling parameters and standard uptake values (SUV) was interrogated with Pearson product-moment correlation coefficients. All statistical analyses were performed with GraphPad Prism 7.0 and Stata 13.1.

## Results

### ***[***^***18***^***F]FEOBV distribution***

[^18^F]FEOBV administration was well tolerated without adverse events*.* The image-derived input function from the aorta lumen peaked at $\sim$ 30 s post injection followed by a rapid decline until $\sim$ 1 min post injection, and then a slow decline throughout the rest of the scan period (Fig. 1). The fitted unmetabolized parent [^18^F]FEOBV was 54% of total activity in venous blood at 30 min, and 30% at 60 min post injection. The average [^18^F]FEOBV fraction in plasma was 0.69, i.e., 31% of tracer was bound to red blood cells (or white blood cells).

**Fig. 2:**
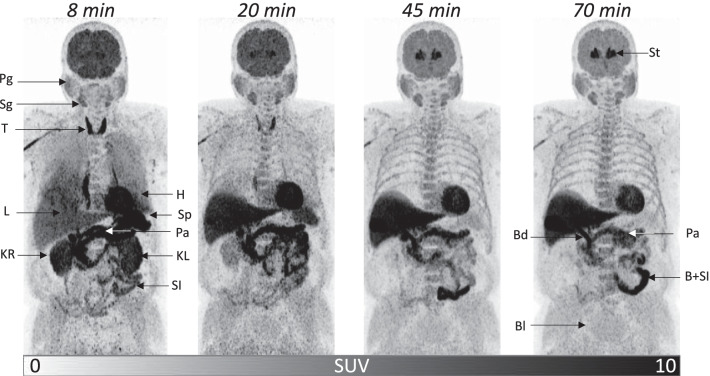
Maximum intensity projection images of whole-body [^18^F]FEOBV distribution at 8, 20, 45, and 70 min post injection. Bd, bile duct; Bl, urinary bladder; B + SI, bile in small intestine; H, heart; KL, left kidney; KR, right kidney; L, liver; Pa, pancreas; Pg, parotid gland; Sg, submandibular gland; SI, small intestine; Sp, spleen; St, striatum; T, thyroid

**Fig. 3:**
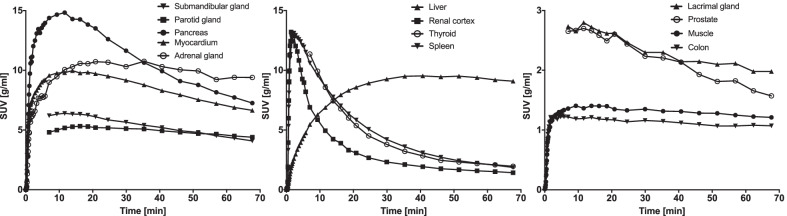
Mean SUV time-activity curves for all organs investigated. Some organs only have data from 6 to 70 min post injection. Note that the thyroid, spleen and renal cortex show similar rapid washout, whereas all other organs display much slower washout

Most visceral organs showed initial high uptake of [^18^F]FEOBV with varying rates and degrees of washout (Figs. 2, 3). Pancreas, myocardium, adrenal gland, muscle, colon, salivary glands, lacrimal gland, and prostate showed relatively slow washout. Spleen, renal cortex and thyroid exhibited faster washout. Liver signal reached a plateau at $\sim$ 30 min post injection. Lung tissue showed a high initial signal with fast washout (not analyzed). The signal was highest in the posterior parts compatible with pooled blood, as subjects were in supine position. Because of this, meaningful esophageal data could not be obtained. In some subjects, small high-uptake areas were observed in the suboccipital and paraclavicular regions, colocalizing with fat on CT, thus probably reflecting tracer accumulation in brown fat tissue. We observed early secretion of [^18^F]FEOBV to the stomach lumen ($\sim$ 6 min post injection), which was further transported to the duodenum. High signal was observed in the biliary ducts after $\sim$ 15 min and in the gallbladder after $\sim$ 20 min. This signal probably reflects biliary secretion of [^18^F]FEOBV (and metabolites), and was visible in the small intestine after $\sim$ 20 min (Fig. 2). The rapid biliary excretion prevented extraction of meaningful data from the small intestine in the present study. Bile was not observed in the colon within the scan period in any of the subjects. Activity was observed in the urinary bladder after $\sim$ 20 min. Some tracer was retained in the veins of the injected arm. In the last time frame, the injected arm contained on average 1.3 MBq (range: 0.1–5.2 MBq) more than the contralateral arm. Thus, the tracer itself may stick to the vascular wall despite generous administration of saline after injection.

### Kinetic model selection

We explored three non-linear models; 1TCM, reversible 2TCM, and irreversible 2TCM. All three models were fitted to TACs of seven different organs available for full kinetic modelling (pancreas, myocardium, spleen, renal cortex, adrenal gland, colon, and muscle), yielding a total of 315 fits (in total 105 VOIs). Assessed by lowest Akaike score, the 1TCM performed best in 34 (32%), the 2TCM in 26 (25%) and the irreversible 2TCM in 45 (43%) VOIs (Additional file 6: Table S2). In 19 fits, the irreversible 2TCM generated extremely low k_3_ values, approximating zero in adrenal gland, pancreas, myocardium, spleen, and muscle. In these cases the 1TCM performed best. The k_3_ coefficient of variation (CoV) ranged between 63 and 380% in these organs while, in contrast, the 1TCM generated robust fits of all organs with CoV ranging from 23 to 39% of the V_t_ estimate. Therefore, we considered the 1TCM to be the most robust non-linear model for [^18^F]FEOBV kinetic modeling of peripheral organs. Kinetic parameters from the 1TCM and V_t_ estimations from Logan plots are presented in Table 1. Fitted V_0_ values were low in adrenal gland, pancreas, and muscle. Therefore, based on blood- and organ volume computations [25], additional fits with constrained *V*_0_ were performed for these three organs (*V*_0_ = blood volume in organ/organ volume). *V*_t_ increased in all *V*_0_-constrained fits (Table 1). The *V*_0_-fixed data did not change the significant linear relationship between *V*_t_ and SUV for these organs (see below). Kinetic parameters for 2TCM and irreversible 2TCM are presented in Additional files 4 and 5: Table 1A + B. Overview of best-fit-models in different organs is presented in Additional file 6: Table 2. We observed a statistically significant correlation between age and myocardium SUV (*r* = 0.60, *P* = 0.019), i.e., increasing myocardium SUV with age. A similar trend was observed for age and myocardium *V*_t_ from the 1TCM (*r* = 0.45, *P* = 0.09). This trend was driven by a significant correlation between age and *k*_2_ (*r* = − 0.57, *P* = 0.026), as no correlation was present between age and *K*_1_ (*r* = 0.05, *P* = 0.86). No other organs displayed age effects.

**Table 1:** Kinetic parameter estimates from the 1-tissue compartment model and Logan plot

Organ	*V*_0_	*K*_1_	*k*_2_	*V*_t_	CoV	AIC	Logan V_t_	CoV	AIC
Adrenal gland	0.01 (0.001–0.08)	0.59 (0.39–0.62)	0.019 (0.007–0.021)	33.1 (30.0–55.9)	39%	76 (19)	29.9 (22.4–33.9)	29%	85 (13)
*- fixed V*_*0*_	*0.23*	*0.73 (0.58–0.77)*	*0.018 (0.006–0.021)*	*45.2 (35.7–83.1)*	*47%*	*91(13)*	–		
Pancreas	0.0035 (0.00023–0.051)	1.00 (0.77–1.17)	0.038 (0.033–0.046)	24.3 (18.5–31.6)	39%	29 (26)	23.6 (19.2–32.2)	30%	106 (17)
*- fixed V*_*0*_	*0.24*	*1.20 (1.00–1.49)*	*0.038 (0.032–0.045)*	*30.0 (23.0–41.1)*	*32%*	*49 (23)*	–		
Myocardium	0.13 (0.07–0.16)	0.63 (0.54–0.78)	0.027 (0.023–0.036)	24.3 (17.8–32.0)	34%	− 20 (28)	21.9 (16.9–27.0)	29%	94 (39)
Spleen	0.17 (0.11–0.25)	1.39 (1.20–1.72)	0.12 (0.10–0.16)	9.72 (9.15–12.7)	26%	2 (28)	9.01 (8.03–10.5)	20%	105 (43)
Renal cortex	0.17 (0.14–0.22)	1.73 (1.25–1.84)	0.25 (0.23–0.27)	6.06 (5.25–7.76)	23%	68 (53)	6.17 (5.41–7.23)	17%	119 (39)
Muscle	0.0004 (0.00002- 0.004)	0.049 (0.04–0.09)	0.012 (0.009–0.016)	4.20 (3.73–5.26)	34%	59 (30)	4.22 (3.03–5.16)	44%	87 (18)
*- fixed V*_*0*_	*0.027*	*0.050 (0.04–0.10)*	*0.01 (0.009–0.014)*	*4.48 (3.92–5.51)*	*28%*	*87 (20)*	–		
Colon	0.027 (0.015–0.033)	0.078 (0.063–0.1)	0.030 (0.021–0.041)	2.64 (1.94–3.51)	37%	97 (16)	3.51 (2.61–4.2)	29%	82 (10)

Values are presented as median (interquartile range) or mean (standard deviation)

*V*_0_ = blood volume fraction [ml/ccm]; *K*_1_ = uptake rate constant [ml/ccm/min]; *k*_2_ = washout rate constant [1/min]; *V*_t_ = volume-of-distribution [ml/ccm]. CoV, coefficient of variation for *V*_t_ estimates (mean/standard deviation); AIC, akaike information criterion. Due to low fitted *V*_0_ values in pancreas, adrenal gland, and muscle, we performed additional fits with fixed *V*_0_

**Table 2:** SUV 50–70 min post injection for all organs

Organ	SUV median (IQR)
Liver	9.2 (7.1–11.0)
Adrenal gland	9.1 (8.0–12.0)
Pancreas	7.0 (6.3–8.9)
Myocardium	7.0 (6.0–7.5)
Parotid gland	4.3 (3.6–5.1)
Submandibular gland	4.1 (3.5–5.4)
Spleen	2.2 (1.9–2.5)
Thyroid	2.1 (1.7–2.4)
Lacrimal gland	2.0 (1.6–2.5)
Prostate	1.6 (1.2–2.2)
Renal cortex	1.5 (1.3–1.7)
Muscle	1.1 (1.0–1.3)
Colon	1.1 (0.8–1.4)

### Linear correlation between V_t_ and SUV

**Fig. 4:**
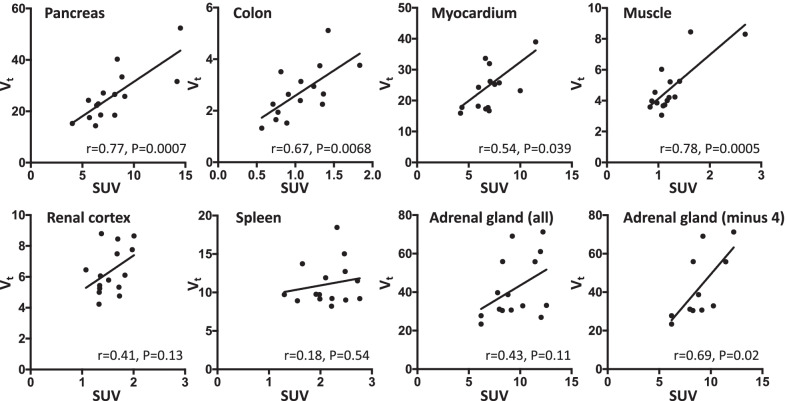
Total volume-of-distribution (*V*_t_) from 1-tissue compartment model versus SUV values from 50 to 70 min post injection. Data are shown with best fitted line (least squares) for visual purpose

The linear relationship between V_t_ from the 1TCM and SUV (average SUV from $\sim$ 50 to 70 min post injection) is shown in Fig. 4. There were significant correlations between *V*_t_ and SUV for pancreas (*r* = 0.77, *P* = 0.0007), colon (*r* = 0.67, *P* = 0.0068), muscle (*r* = 0.78, *P* = 0.0005), and myocardium (*r* = 0.54, *P* = 0.039). There was a trend towards a linear relationship in the adrenal gland (*r* = 0.43, *P* = 0.11) and renal cortex (*r* = 0.41, *P* = 0.13). In four subjects, the adrenal gland was only partly included in the field-of-view. Exclusion of these four data points resulted in a significant linear correlation in the remaining 11 adrenal gland data points (*r* = 0.69, *P* = 0.02). No correlation was observed in the spleen (*r* = 0.18, *P* = 0.54). The linear relationship between V_t_ from Logan plot and SUV was similar, except for the colon where the linear relationship was not significant (*r* = 0.5, *P* = 0.06). Nearly identical correlations were achieved with SUV from 50–60 min and from 60–70 min post injection (data not shown). SUVs for all organs are shown in Table 2. A very strong correlation between *V*_t_ derived from 1TCM and Logan plot was observed in pancreas, spleen, myocardium, renal cortex, and muscle (*r* > 0.85, *P* < 0.0001). Less strong, but significant, correlations were observed between 1TCM and Logan plot *V*_t_ in colon (*r* = 0.67, *P* = 0.007) and adrenal gland (*r* = 0.74, *P* = 0.0035).

## Discussion

In the present study we describe the distribution of [^18^F]FEOBV in 13 peripheral organs of healthy human subjects. We tested three non-linear kinetic models of the pancreas, renal cortex, spleen, myocardium, muscle, adrenal gland, and colon. The 1TCM generally performed best, with robust fits of *V*_t_. Although *V*_t_ does not represent specific binding of [^18^F]FEOBV, it reflects the degree of tracer being concentrated in tissue, which is interpretated as an indirect measure of VAChT density in the present study. The fitted *V*_0_ was lower than expected in adrenal gland, pancreas, and muscle. Therefore, we reanalyzed these data with constrained *V*_0_ which increased *V*_t_ by 7% in muscle, 23% in pancreas, and 37% in adrenal gland. Hence, the original fits may have underestimated the true *V*_t_ in these organs. Colon V_0_ was low, but the colon VOI unavoidably contains feces without blood supply or [^18^F]FEOBV binding. Determination of an intestinal-wall-only VOI is practically impossible. Thus, if the actual colon wall occupies 10% of the colon VOI, it would then translate into a *V*_0_ of $\sim$ 30%, in line with calculated estimates of colon-wall blood volume fraction [25]. V_0_ in other organs seemed reliable.

We could not determine free fraction of [^18^F]FEOBV in plasma due to high centrifugal filter retainment. This occurred even when [^18^F]FEOBV was dissolved in a buffer solution. To our knowledge, no other studies have investigated the free fraction of [^18^F]FEOBV. Therefore, the free fraction was conservatively set to 1, which underestimates *V*_t_ by an unknown magnitude.

As the input function was extracted from whole blood PET data in the aorta lumen, binding of [^18^F]FEOBV to red blood cells was measured. We found that 69% of [^18^F]FEOBV was located in plasma (i.e., not bound to red blood cells), which was used to correct the input function in all subjects. Differences between individual subjects could have affected model parameters. Also, hematocrit differences between arterial blood and venous blood could have influenced this fraction (as hematocrit was measured in venous blood). However, one study showed that hematocrit is only 3% higher in venous blood compared to arterial blood [26]. Thus, this difference is not considered significant in the present study. Overestimation of the aorta input function is a potential risk in the image-derived input model, as spill-in from surrounding hot organs (lung, heart, liver) may occur. However, great care was taken to avoid lung-spill-in in the first part of the scan, where the lung signal was high. Spill-in from heart or liver would primarily be a problem caused by subject movement. This would have led to a significant activity increase on the aorta TAC, but this was not observed during visual inspection of the data. In support, spill-in was not observed in an [^18^F]FDG study comparing imaged-derived input from aorta to arterial blood sampling (Additional file 3: Fig. S2). The 1TCM *V*_t_ estimates were compared to *V*_t_ computed through a linearized approach (Logan plot). The two approaches generated comparable estimates of *V*_t_, except for a slightly lower Logan *V*_t_ in the adrenal gland and myocardium, and a slightly higher *V*_t_ in the colon (Table 1). Logan plots may underestimate the true *V*_t_ compared to non-linear models, and the degree of negative bias depends on the noise and magnitude of the true *V*_t_ [27]. Therefore, the hotter and more noisy data from particularly the adrenal gland may have caused an underestimation of *V*_t_ in the Logan plot. However, the uncertainties regarding the input function (mentioned above) may have caused and overestimation in 1TCM. Thus, it is unclear whether 1TCM or Logan plot predicts the most reliable estimate of the *V*_t_.

The [^18^F]FEOBV signal in each organ has different origins and reflects different cholinergic functions. Microvasculature (but not macrovasculature) in visceral organs, except kidney, liver, and spleen, receive VAChT positive parasympathetic neurons [28].

*The pancreas* exhibited high [^18^F]FEOBV accumulation with a relatively slow washout, suggestive of high VAChT density. Indeed, pancreatic VAChT containing neurons are abundant; vagal preganglionic parasympathetic neurons project to postganglionic parasympathetic neurons that are often clustered in intrapancreatic interlobular ganglia [15, 28]. These cholinergic neurons regulate the exocrine- and endocrine (islet) structures of the pancreas through acetylcholine release from dense varicosities along the axon, and not through well-defined pre- and postsynaptic elements as in other organs [29, 30]. In addition to the parasympathetic neurons, human alpha-cells in the islets also stain for VAChT [31]. The fractional [^18^F]FEOBV signal contribution from alpha-cell uptake in the islets is impossible to estimate. Islets show intense staining in immunohistochemistry studies, but occupy less than 5% of total pancreatic volume, and alpha-cells only account for $\sim$ 20% of islet cells [32]. Another human in vivo imaging study using [^11^C]-donepezil PET estimated an almost eight times higher *V*_t_ in pancreas than in our study [33]. This difference may be generated by [^11^C]donepezil binding to exocrine acinar cells, which show very low VAChT staining in rat immunohistochemistry studies [34].

*Myocardium* of the left ventricle showed high [^18^F]FEOBV binding similar to that of the pancreas. VAChT-immunoreactive neurons are discernable in ventricles, but higher density is observed in atria [15, 28]. However, cardiomyocytes also possess a full cholinergic machinery including VAChT [35]. Cardiomyocyte-derived acetylcholine probably protects the heart in stressful situations through paracrine signaling [36]. Therefore, [^18^F]FEOBV PET of myocardium could be relevant in cardiac diseases with low cholinergic activity, e.g., chronic heart failure. One PET study has shown that [^18^F]FEOBV is suitable for measuring cardiac cholinergic activity in humans [24]. In that study, Logan plots, 1TCM, and 2TCM were performed showing little variation in *V*_t_ (average $\sim$ 3 ml/ccm). In the present study, median myocardium *V*_t_ was 23.4 ml/ccm using 1TCM and 21.9 ml/ccm using Logan plot. The lower *V*_t_ found by Saint-Georges et al. may partly be explained by lack of metabolite correction, which would result in a major underestimation of *V*_t_ as [^18^F]FEOBV is rapidly metabolized. Also, differences in extraction of image-derived input function (we used the aorta lumen VOI for this post hoc analysis) and the left ventricular myocardium time-activity curve may contribute to the large inconsistency, but this information has not been published in detail [24]. Our *V*_t_ estimates decreased approximately 50% when we analyzed our data similarly to that study, i.e., 25 min TAC and no metabolite correction but were still approximately four times higher. However, the mean age was also substantially higher in our study (72 years vs. 37 years). Although we did not find a statistically significant correlation with age for the V_t_ estimate, we did see a trend (*r* = 0.45, *P* = 0.09). The slope of the best fitted line was 0.49 suggesting that *V*_t_ increases 0.49 every year. This is probably highly overestimated as the y-intercept was − 10. Still, this points to a potential dramatic age influence on the myocardial [^18^F]FEOBV uptake, and could account for some of the between-study discrepancy. Interestingly, the trend was driven by a statistically significant negative correlation between age and *k*_2_, and not *K*_1_. This indicates that it is *not* the extraction of [^18^F]FEOBV from blood to tissue that is altered with age—which could be influenced by perfusion. Rather, it is the rate of [^18^F]FEOBV being transported from the myocardium back to blood, indicative of higher specific binding. We previously found a similar age effect for acetylcholinesterase in an [^11^C]donepezil PET study [37]. We argued that it could be caused by sigma-1 receptor upregulation. However, if VAChT is also upregulated with age, it suggests that the entire cardiomyocyte-derived cholinergic machinery may be upregulated in the aging heart.

*The adrenal gland* displayed the highest [^18^F]FEOBV signal of all investigated organs in the present study. The adrenal gland is innervated by preganglionic sympathetic neurons from the intermediolateral cell column in the thoracic spine [28]. Two immunohistochemistry studies in rats showed intense VAChT signal in medullary nerve fibers, but no signal in medullary cells or in the cortex [15, 38]. This suggests, that the intense signal seen in the present study is generated solely by the cholinergic preganglionic neurons in the medulla. Since the medulla is not visible on CT, we normalized the total PET signal to the anatomical volume of the entire adrenal gland (including cortex). This may have severely underestimated the V_t_ as the human adrenal medulla only occupies about 10% of total adrenal gland volume [39].

*The colon* showed low [^18^F]FEOBV signal compared to other organs, but also a slow washout. As mentioned above, the colon PET signal is normalized to a CT-derived colon volume that include feces (but exclude air). This circumstance underestimates the true colonic [^18^F]FEOBV signal—probably by at least an order of magnitude. In the human colon, more than 50% of enteric neurons in the myenteric and submucosal plexi stain for ChAT [40]. ChAT colocalizes almost completely with VAChT [12]. Preganglionic parasympathetic neurons originating from the dorsal motor nucleus of the vagus and sacral intermediolateral cell column, project to both myenteric and, to a lesser degree, submucosal neurons [41]. Anterograde labeling studies from the dorsal motor nucleus of the vagus have shown that the intensity of vagal parasympathetic innervation decreases in the anal direction of the gastrointestinal tract [42]. It is likely that the primary source of colon [^18^F]FEOBV binding is enteric cholinergic neurons, with a lower contribution from vagal parasympathetic projections. In support, bilateral vagotomy in rats elicit decreased VAChT staining density in the upper GI tract but not in the colon (N Van Den Berge—unpublished data).

*Spleen and renal cortex* displayed similar [^18^F]FEOBV kinetics with an initial peak followed by rapid washout, although a bit slower in the spleen resulting in a higher *V*_t_ (9.72 vs. 6.06 ml/ccm) (Fig. 3). This suggests an absence of high VAChT densities in these organs. Cholinergic innervation of the spleen has been debated as early studies showed cholinergic markers in lysate of mammal spleen tissue (reviewed in [43]). However, there is strong evidence that no cholinergic neurons supply the spleen [44–47]. Similarly in the kidney, the presence of cholinergic innervation has been debated as retrograde labeling studies have failed to show parasympathetic origin of neuronal structures [48–50]. Instead, the source of cholinergic markers in kidney were proposed to be of non-neuronal origin [48]. The higher *V*_t_ in spleen than renal cortex may be explained by [^18^F]FEOBV binding to immune- and red blood cells in spleen parenchyma [51].

*Resting muscle* showed low [^18^F]FEOBV uptake, but barely any washout*.* The neuromuscular endplate from somatic motor neurons shows intense VAChT immunoreactivity [15]. Furthermore, the muscular microvasculature also receives VAChT-containing nerve fibers. Thus, the muscular [^18^F]FEOBV signal probably constitute both somatic- and parasympathetic efferent nerve fiber binding. One subject had significantly higher signal than average (Fig. 4). It could be speculated that this patient had muscular inflammation, since [^18^F]FEOBV is also known to show some binding to immune cells [52].

*The liver* signal was not investigated with kinetic modelling as the rapid increase in signal is caused by tracer metabolization in hepatocytes (and further secretion into bile). In addition, no VAChT neurons are visible in rat liver [15, 28].

*Salivary- and lacrimal glands* showed moderate [^18^F]FEOBV signal, with relatively slow washout. The glands contain postganglionic parasympathetic neurons from the otic, submandibular, and pterygopalatine ganglia (innervated by preganglionic parasympathetic neurons from the salivatory nuclei). They can be macroscopically visualized with VAChT immunostaining in the vicinity of exocrine acini, ducts, myoepithelium, and microvasculature [15, 28]. Thus, [^18^F]FEOBV signal in salivary- and lacrimal gland tissue may be of neuronal origin only. It is unknown whether the tracer is secreted into the saliva and lacrimal gland fluids.

*The thyroid* showed initial intense [^18^F]FEOBV signal, followed by a rapid washout, suggestive of low VAChT density. Two human studies showed AChE containing neurons in the thyroid [53, 54], but no studies using more specific markers (VAChT or ChAT) have been published. In chicken thyroid, adrenergic nerves outnumber those with AChE [55]. Similarly, nuclide imaging of adrenergic nerves shows higher thyroid binding compared to our study (although a direct comparison has not been made) [56].

*The prostate* showed moderate [^18^F]FEOBV uptake with relatively slow washout. VAChT containing neurons are abundant in all parts of prostatic tissue [28, 57]. Thus, [^18^F]FEOBV retainment is probably primarily of preganglionic and postganglionic parasympathetic origin.

*Bone tissue,* probably mainly restricted to the bone marrow*,* also showed relatively high [^18^F]FEOBV uptake (Fig. 2). Sparse evidence suggest that cholinergic (probably sympathetic) neurons innervate the bone marrow in rodents [58, 59]. One study reported evidence parasympathetic innervation of the metaphysis in rat [60]. However, cartilage, chondroblasts and mesenchymal stem cells also contain VAChT in rats [61], so the potential neuronal contribution to tracer uptake is unknown, but may be small.

We found a significant linear association between *V*_t_ and SUV in most organs (Fig. 4). In four subjects, only part of the adrenal gland was included in the first 6 min of the PET scan. The vicinity of the VOI to the field-of-view border increased noise in TAC, especially in the first part, challenging a reliable *V*_t_ estimation. Indeed, when these four subjects were excluded, a significant relationship between *V*_t_ and SUV emerged.

This study has several limitations. The most important involve assumptions in the input model for kinetic modeling—all of which are mentioned above. In short, image-derived input functions were metabolite corrected based on group-based data, and corrected for red blood cell binding based on two experiments in healthy controls. Thus, interindividual variations in these parameters were not assessed, and may have affected the parameter estimates. Furthermore, our sample size was only modest, but large enough to show the linear relationship between *V*_t_ from kinetic modeling and the simple SUV. Potential tissue metabolism was not investigated in the present study, only blood metabolism. This could have led to overestimations of organ *V*_t_. We only acquired whole-body data from 0 to 70 min post injection. Later time points could potentially yield more stable and consistent modeling parameters. However, due to the biliary secretion it is unlikely to get meaningful colon data at later time points.

## Conclusion

We believe that [^18^F]FEOBV PET is a suitable method for measuring VAChT density in peripheral organs. Studying the neuronal contribution to [^18^F]FEOBV signal may be of particular interest in neurodegenerative diseases such Parkinson’s disease and Dementia with Lewy bodies, but also other diseases showing autonomic denervation including diabetic neuropathy. Non-neuronal VAChT containing cells in heart and pancreas may serve as important research targets in heart diseases and diabetes. Simple static SUVs between 50- and 70 min post injection may replace full kinetic modeling for adrenal gland, pancreas, myocardium, muscle, and colon. This would be favorable for participants, as the scan duration is significantly reduced.

## Supplementary Information

**Additional file 1. **Description of radiochemical analyses.

**Additional file 2. Figure S1: **Examples of volume-of-interest definitions from all organs in the study. a: Pancreas; b: Liver; c: Spleen; d: Myocardium; e: Muscle; f: Renal cortex; g: Aorta; h: Thyroid; i: Lacrimal gland; j: Prostate; k: Adrenal gland (CT); l: Adrenal gland (PET/CT); m: Colon (CT); n: Colon (PET/CT); o: Parotid gland (CT); p: Parotid gland (PET/CT); q: Submandibular gland (CT); r: Submandibular gland (PET/CT). CT and PET/CT images are shown for the same slice (k+l, m+n, o+p, q+r). CT-derived VOIs are also displayed on the PET/CT image to show the slightly larger PET volume than anatomical CT-derived volume. PET signal is scaled to the level used in analyses of each organ, i.e., PET signal scale differs between images.

**Additional file 3. Figure S2: **Arterial blood 18F-FDG time-activity curves (TACs) obtained from arterial blood samples (black) and auto-generated aorta-VOI (red). The curves are practically identical.

**Additional file 4. Table S1A: **Kinetic parameter estimates from the 2-tissue compartment model. Values are presented as median (interquartile range) or mean (standard deviation). V0 = blood volume fraction [ml/ccm]; K1 = uptake rate constant [ml/ccm/min]; k2 = washout rate constant [1/min]; k3 = rate of tracer association to VAChT [1/min]; k4 = rate of tracer dissociation to VAChT [1/min]; CoV = Coefficient of variation for Vt estimates (mean/standard deviation); AIC = Akaike information criterion. *One fit failed.

**Additional file 5. Table S1B: **Supplementary Table 1B. Kinetic parameter estimates from the irreversible 2-tissue compartment model. Values are presented as median (interquartile range) or mean (standard deviation). V0 = blood volume fraction [ml/ccm]; K1 = uptake rate constant [ml/ccm/min]; k2 = washout rate constant [1/min]; k3 = rate of tracer association to VAChT [1/min]. CoV = Coefficient of variation for k3 estimates (mean/standard deviation); AIC = Akaike information criterion. *Five k3 values approximated 0.

**Additional file 6. Table S2: **Best-fit-models in different organs. Comparison of 1-tissue-, 2-tissue- and irreversible 2-tissue compartment models.

## Data Availability

Data will be provided by the corresponding author upon reasonable request.
