# Supplementary material for: In vivo vesicular acetylcholine transporter density in human peripheral organs: an [18F]FEOBV PET/CT study
Source: EJNMMI Res. 2022 Apr 1;12:17. doi: 10.1186/s13550-022-00889-9 (PMC8975951; doi:10.1186/s13550-022-00889-9)
Supplement: Supplementary file 1 — Additional file 1. Description of radiochemical analyses. [file 13550_2022_889_MOESM1_ESM.docx]

**Supplementary material**

*[^18^F]FEOBV tracer preparation*

[^18^F]FEOBV synthesis was performed using a General Electric (GE) TRACERLab FXFN synthesis module. A GE PETtrace cyclotron equipped with a high-yield fluorine-18 target, produced [^18^F]Fluoride, which was delivered in a bolus of ^18^O-H_2_O to the synthesis module, and subsequently trapped on a QMA-Light sep-pak cartridge to remove ^18^O-H_2_O. Fluoride was eluted from the QMA Sep-Pak cartridge using 0.6 ml Kryptofix eluent solution (elution solution, reagent 1.1.1.). Water was removed by heating the reactor containing the 600 μl eluent solution and [^18^F]fluoride to 110°C. In addition, vacuum was applied with a flow of high purity helium directed through the reactor. A small portion of acetonitrile (300 μl) was added to the reactor to facilitate the evaporation of water by azeotropic distillation.

FEOBV (precursor) was dissolved in 1 ml of DMSO, and added to the dried [^18^F]fluoride at 120°C, followed by a labeling time of 10 min. Next, the reaction mixture was cooled (50°C), diluted with H_2_O (3 ml), and purified by semi-preparative HPLC (column: Zorbax XDB-C18, 9.4 x 250 mm, 5 μm; mobile phase: 34% ethanol 70 mM Na_2_HPO_4_; flow rate: 5 ml/min; UV: 220 nm). The product peak (t_R_ = 9-11 min) was collected and diluted with a large volume of sterile water (70 ml). The diluted fraction was then passed slowly through a C8 Sep-Pak cartridge and purified by solid phase extraction. The single-use C8 Sep-Pak was preconditioned by 10 ml sterile ethanol and 10 ml sterile water. The produced [^18^F]FEOBV was retained on the cartridge, and washed with 10 ml of sterile water. Then, the product was eluted with 1 ml of ethanol followed by 9 ml of saline for formulation. The formulated product was finally passed through a sterilizing filter (0.22 μm) to the sterile product vial. Radiochemical purity was > 95%.

*Estimation of [^18^F]FEOBV binding in plasma (not bound to red blood cells)*

First, 20 $\mu$l tracer (approximately 10kBq) was added to 10 ml blood drawn into EDTA tubes from two healthy volunteers. At time 0, 5, 15, 30, 45, 60 min after tracer administration, the EDTA tubes were gently turned, and 1.5 ml whole blood was drawn into heparinized tubes. From these sample, 100 $\mu$l whole blood was drawn for activity measurement in a well counter (*AMG, Hidex*). Further 500 $\mu$l whole blood was centrifugated (3 min x 12540 rpm), and subsequently 100 $\mu$l plasma was drawn for activity measurement. Raw counting numbers were used for statistics, and the fraction of [^18^F]FEOBV in plasma was calculated as: (plasma counts*(1-haematocrite))/whole blood counts. During the entire experiment, the original blood samples with tracer were kept at 37$℃$.

*Determination of parent [^18^F]FEOBV in venous blood samples*

Venous blood samples were drawn at 5, 15, 30, 45, and 60 min post injection. Then, 500 µL plasma was mixed with 500 μL acetonitrile acid to precipitate plasma proteins. The supernatant was analyzed and fractionated by HPLC (Phenomenex Luna C18(2) 10 µm 250x10 mm, ACN:70 mM NaH_2_PO_4_, 50:50, 5 ml/min) after centrifugation (5 min × 13.000 rpm). Detection consisted of serial ultraviolet detection (λ = 254 nm) and gamma detection. Activity concentration was measured in a well counter (*AMG, Hidex*).
