# Supplementary figures and images for: In vivo vesicular acetylcholine transporter density in human peripheral organs: an [18F]FEOBV PET/CT study
Source: EJNMMI Res. 2022 Apr 1;12:17. doi: 10.1186/s13550-022-00889-9 (PMC8975951; doi:10.1186/s13550-022-00889-9)

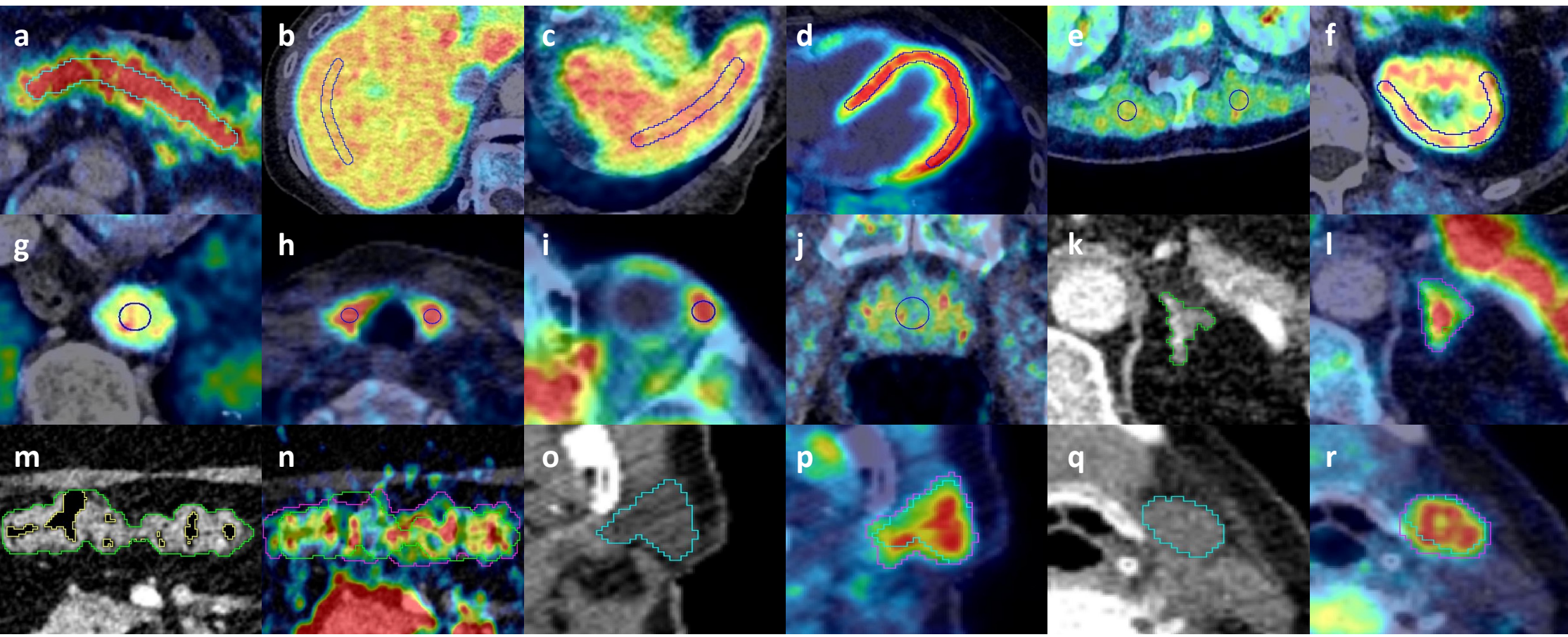

Supplement: Supplementary file 2 — Additional file 2. Figure S1: Examples of volume-of-interest definitions from all organs in the study. a: Pancreas; b: Liver; c: Spleen; d: Myocardium; e: Muscle; f: Renal cortex; g: Aorta; h: Thyroid; i: Lacrimal gland; j: Prostate; k: Adrenal gland (CT); l: Adrenal gland (PET/CT); m: Colon (CT); n: Colon (PET/CT); o: Parotid gland (CT); p: Parotid gland (PET/CT); q: Submandibular gland (CT); r: Submandibular gland (PET/CT). CT and PET/CT images are shown for the same slice (k+l, m+n, o+p, q+r). CT-derived VOIs are also displayed on the PET/CT image to show the slightly larger PET volume than anatomical CT-derived volume. PET signal is scaled to the level used in analyses of each organ, i.e., PET signal scale differs between images. [file 13550_2022_889_MOESM2_ESM.pdf]

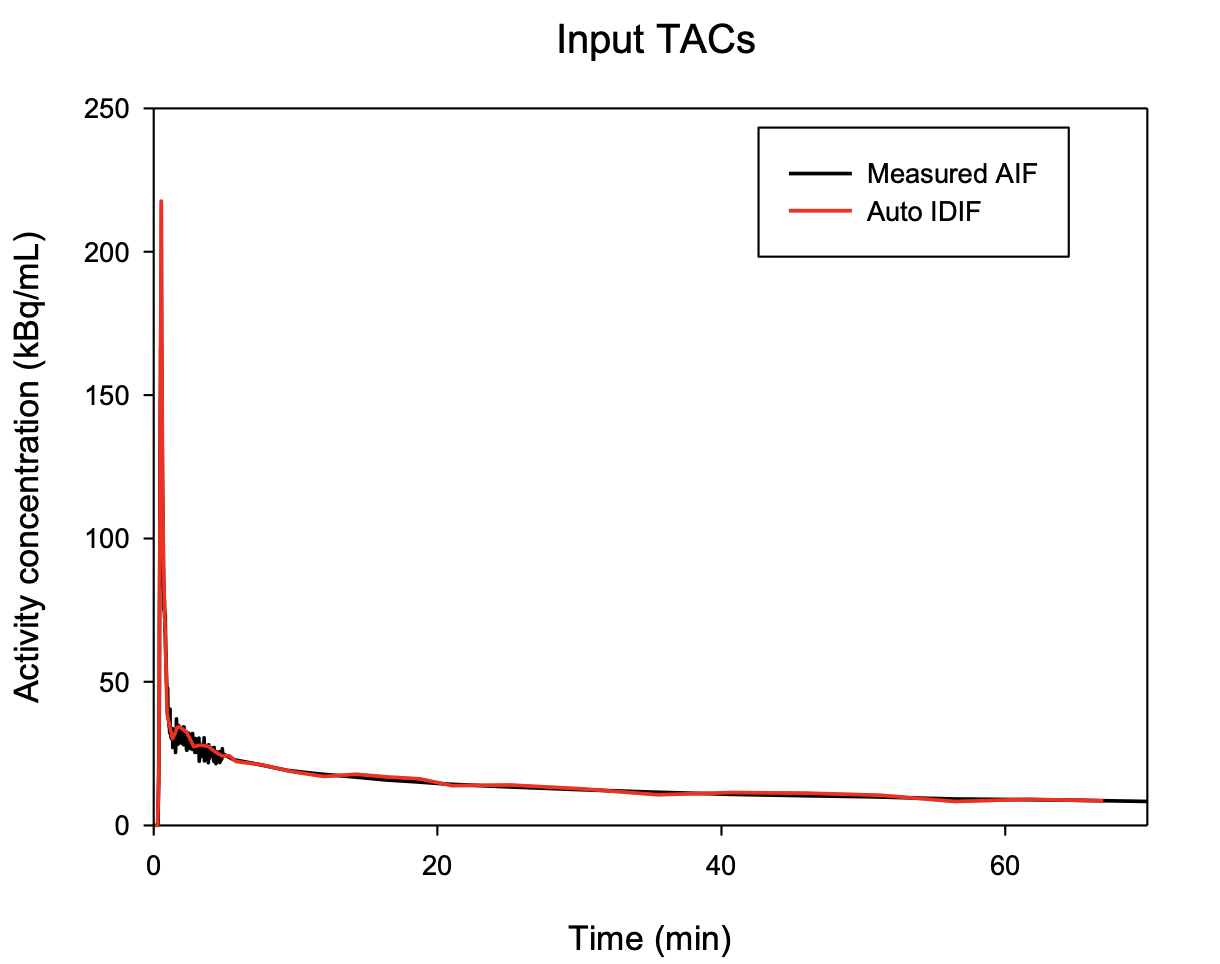

Supplement: Supplementary file 3 — Additional file 3. Figure S2: Arterial blood 18F-FDG time-activity curves (TACs) obtained from arterial blood samples (black) and auto-generated aorta-VOI (red). The curves are practically identical. [file 13550_2022_889_MOESM3_ESM.jpg]
