# Supplementary material for: In vivo vesicular acetylcholine transporter density in human peripheral organs: an [18F]FEOBV PET/CT study
Source: EJNMMI Res. 2022 Apr 1;12:17. doi: 10.1186/s13550-022-00889-9 (PMC8975951; doi:10.1186/s13550-022-00889-9)
Supplement: Supplementary file 4 — Additional file 4. Table S1A: Kinetic parameter estimates from the 2-tissue compartment model. Values are presented as median (interquartile range) or mean (standard deviation). V0 = blood volume fraction [ml/ccm]; K1 = uptake rate constant [ml/ccm/min]; k2 = washout rate constant [1/min]; k3 = rate of tracer association to VAChT [1/min]; k4 = rate of tracer dissociation to VAChT [1/min]; CoV = Coefficient of variation for Vt estimates (mean/standard deviation); AIC = Akaike information criterion. *One fit failed. [file 13550_2022_889_MOESM4_ESM.docx]

**Supplementary Table 1A.** Kinetic parameter estimates from the 2-tissue compartment model.

| Organ | V_0_ | K_1_ | k_2_ | k_3_ | k_4_ | V_t_ | CoV | AIC |
| --- | --- | --- | --- | --- | --- | --- | --- | --- |
| Adrenal gland* | 0.0064 (0.0011-0.052) | 0.59 (0.37-0.65) | 0.021 (0.017-0.046) | 0.29 (0.005-1.9) | 2.1 (0.13-7.71) | 41.4 (31.1-70.3) | 374% | 76 (18) |
| Pancreas* | 0.007 (0.00003-0.06) | 1.01 (0.82-1.2) | 0.047 (0.036-0.056) | 0.0038 (0.0016-0.015) | 0.037 (0.000-1.75) | 2*10^53^ (26-2*10^85^) | 372% | 27 (27) |
| Myocardium | 0.12 (0.05-0.17) | 0.63 (0.54-0.8) | 0.029 (0.024-0.036) | 0.0015 (3*10^-5^-0.0032) | 0.0019 (0.000-0.015) | 26.6 (23.5-3*10^6^) | 344% | -21 (31) |
| Spleen | 0.14 (0.021-0.21) | 1.36 (1.18-1.65) | 0.18 (0.11-0.67) | 0.026 (0.0035-0.099) | 0.20 (0.036-0.25) | 9.57 (2.15-12.63) | 297% | 54 (117) |
| Renal cortex | 0.15 (0.088-0.19) | 1.71 (1.17-1.81) | 0.33 (0.29-0.43) | 0.066 (0.014-0.088) | 0.15 (0.034-0.35) | 7.25 (5.1-7.77) | 43% | 74 (93) |
| Muscle* | 8*10^-6^ (0.000-0.0006) | 0.05 (0.04-0.14) | 0.02 (0.011-0.059) | 0.0098 (0.0028-0.042) | 0.022 (0.000-0.72) | 6.7 (4.2-4*10^32^) | 374% | 46 (39) |
| Colon | 0.0084 (6*10^-4^-0.017) | 0.10 (0.0840.12) | 0.11 (0.10-0.14) | 0.065 (0.033-0.10) | 0.018 (0.0028-0.029) | 4.9 (3.3-12.1) | 305% | 47 (14) |

*Values are presented as median (interquartile range) or mean (standard deviation). V_0_ = blood volume fraction [ml/ccm]; K_1_ = uptake rate constant [ml/ccm/min]; k_2_ = washout rate constant [1/min]; k_3_ = rate of tracer association to VAChT [1/min]; k_4_ = rate of tracer dissociation to VAChT [1/min]; CoV = Coefficient of variation for V_t_ estimates (mean/standard deviation); AIC = Akaike information criterion. *One fit failed.*
