# Supplementary material for: In vivo vesicular acetylcholine transporter density in human peripheral organs: an [18F]FEOBV PET/CT study
Source: EJNMMI Res. 2022 Apr 1;12:17. doi: 10.1186/s13550-022-00889-9 (PMC8975951; doi:10.1186/s13550-022-00889-9)
Supplement: Supplementary file 5 — Additional file 5. Table S1B: Supplementary Table 1B. Kinetic parameter estimates from the irreversible 2-tissue compartment model. Values are presented as median (interquartile range) or mean (standard deviation). V0 = blood volume fraction [ml/ccm]; K1 = uptake rate constant [ml/ccm/min]; k2 = washout rate constant [1/min]; k3 = rate of tracer association to VAChT [1/min]. CoV = Coefficient of variation for k3 estimates (mean/standard deviation); AIC = Akaike information criterion. *Five k3 values approximated 0. [file 13550_2022_889_MOESM5_ESM.docx]

**Supplementary Table 1B.** Kinetic parameter estimates from the irreversible 2-tissue compartment model.

|  |  |  | |  | |  | | |  |  |  |  |  |  |
| --- | --- | --- | --- | --- | --- | --- | --- | --- | --- | --- | --- | --- | --- | --- |
| Organ | **V_0_** | | **K_1_** | | **k_2_** | | **k_3_** | **CoV** | **AIC** |  |  |  |  |  |
| Adrenal gland | 0.018 (0.000-0.084) | | 0.59 (0.39-0.62) | | 0.020 (0.009-0.027) | | 0.000 (0.000-0.022) | 380% | 75 (17) |  |  |  |  |  |
| Pancreas | 0.0047 (0.0005-0-049) | | 1.01 (0.79-0.18) | | 0.039 (0.037-0.055) | | 0.0023 (0.000-0.0052)* | 94% | 24 (26) |  |  |  |  |  |
| Myocardium | 0.12 (0.05-0.17) | | 0.63 (0.54-0.80) | | 0.030 (0.024-0.038) | | 0.0033 (0.0015-0.0058) | 71% | -33 (27) |  |  |  |  |  |
| Spleen | 0.16 (0.085-0.23) | | 1.39 (1.18-1.72) | | 0.12 (0.11-0.16) | | 0.0013 (0.0006-0.0020) | 63% | -4 (33) |  |  |  |  |  |
| Renal cortex | 0.17 (0.084-0.21) | | 1.67 (1.25-1.84) | | 0.26 (0.24-0.29) | | 0.0038 (0.0025-0.0056) | 45% | 42 (28) |  |  |  |  |  |
| Muscle | 8*10^-5^ (0.000-0.0011) | | 0.05 (0.04-0.10) | | 0.013 (0.010-0.022) | | 0.0072 (0.0016-0.012) | 74% | 37 (23) |  |  |  |  |  |
| Colon | 0.005 (10^-4^-0.019) | | 0.09 (0.08-0.12) | | 0.074 (0.058-0.10) | | 0.025 (0.021-0.034) | 56% | 49 (15) |  |  |  |  |  |

*Values are presented as median (interquartile range) or mean (standard deviation). V_0_ = blood volume fraction [ml/ccm]; K_1_ = uptake rate constant [ml/ccm/min]; k_2_ = washout rate constant [1/min]; k_3_ = rate of tracer association to VAChT [1/min]. CoV = Coefficient of variation for k_3_ estimates (mean/standard deviation); AIC = Akaike information criterion. * Five k_3_ values approximated 0.*
