# Supplementary material for: In vivo vesicular acetylcholine transporter density in human peripheral organs: an [18F]FEOBV PET/CT study
Source: EJNMMI Res. 2022 Apr 1;12:17. doi: 10.1186/s13550-022-00889-9 (PMC8975951; doi:10.1186/s13550-022-00889-9)
Supplement: Supplementary file 6 — Additional file 6. Table S2: Best-fit-models in different organs. Comparison of 1-tissue-, 2-tissue- and irreversible 2-tissue compartment models. [file 13550_2022_889_MOESM6_ESM.docx]

**Supplementary Table 2.** Best-fit-models in different organs. Comparison of 1-tissue-, 2-tissue- and irreversible 2-tissue compartment models.

|  |  | | |  |  |  |  |
| --- | --- | --- | --- | --- | --- | --- | --- |
| Organ | | **1TCM** | **2TCM** | | | | **Irreversible 2TCM** |
| Adrenal gland | | 12 | 2 | | | | 1 |
| Pancreas | | 6 | 0 | | | | 9 |
| Myocardium | | 5 | 1 | | | | 9 |
| Spleen | | 3 | 5 | | | | 7 |
| Renal cortex | | 3 | 8 | | | | 4 |
| Muscle | | 5 | 1 | | | | 9 |
| Colon | | 0 | 9 | | | | 6 |
| Total | | **34 (32%)** | **26 (25%)** | | | | **45 (43%)** |
